# Immune and physiological responses in *Penaeus monodon* to ammonia-N stress: a multi-omics approach

**DOI:** 10.3389/fimmu.2024.1510887

**Published:** 2024-12-10

**Authors:** Zhi Luo, Falin Zhou, Song Jiang, Jianhua Huang, Lishi Yang, Qibin Yang, Jianzhi Shi, Erchao Li, Zhenhua Ma, Yundong Li

**Affiliations:** ^1^ South China Sea Fisheries Research Institute, Chinese Academy of Fishery Sciences, Key Laboratory of South China Sea Fishery Resources Exploitation and Utilization, Ministry of Agriculture and Rural Affairs, Guangzhou, China; ^2^ Laboratory of Aquaculture Nutrition and Environmental Health, School of Life Sciences, East China Normal University, Shanghai, China; ^3^ Key Laboratory of Efficient Utilization and Processing of Marine Fishery Resources of Hainan Province, Sanya Tropical Fisheries Research Institute, Sanya, China; ^4^ Shenzhen Base of South China Sea Fisheries Research Institute, Chinese Academy of Fishery Sciences, Shenzhen, China

**Keywords:** ammonia-N stress, transcriptome, metabolome, shrimp, immune response, oxidative stress

## Abstract

Ammonia-N stress is a significant environmental factor that adversely affects the health and productivity of aquaculture species. This study investigates the effects of ammonia-N stress on the shrimp *Penaeus monodon* through a combination of biochemical, histological, transcriptomic, and metabolomic analyses. Shrimp were exposed to ammonia-N stress for 12 and 96 hours, and key markers of oxidative stress, nitrogen metabolism, immune response, and overall health were assessed. The results showed that prolonged ammonia-N exposure causes significant hepatopancreatic damage, including atrophy and deformation. Transcriptomic analysis revealed significant changes in gene expression related to apoptosis, immune response, and key metabolic pathways, with particular emphasis on the disruption of innate immune signaling and defense mechanisms. Metabolomic analysis identified disruptions in nucleotide turnover, antioxidant defenses, and fundamental metabolic processes. These findings suggest that ammonia-N stress induces a multifaceted stress response in shrimp, involving oxidative stress, immune activation, and metabolic disturbances. Understanding these immune-related and metabolic mechanisms provides valuable insights into the molecular responses of crustaceans to environmental stress, laying the foundation for assessing the ecological risk of ammonia-N and identifying potential immunological biomarkers for monitoring and mitigating its adverse effects in aquaculture systems.

## 1 Introduction

Aquaculture has become a crucial industry, providing high-quality protein and contributing significantly to the global economy (1, 2). It plays a vital role in food security, offering a sustainable source of seafood to meet the increasing demand of the growing human population (3). However, aquaculture faces several challenges that can affect its sustainability and productivity. These challenges include disease outbreaks, water quality management, and environmental pollution (4). Among these, ammonia-N stress is a major concern, as it can lead to severe physiological and metabolic disturbances in aquatic organisms (5, 6). In natural environments, ammonia-N concentrations are typically low, generally not exceeding 5 mg/L. However, due to industrial wastewater discharge, under extreme conditions, ammonia-N concentrations in certain areas can exceed 100 mg/L (7). On the other hand, in modern intensive aquaculture, ammonia-N concentrations often rise rapidly due to high-density stocking and limited water volume, leading to significant ecological and physiological stress (8, 9).

Ammonia-N is a byproduct of organic matter decomposition and excretion by aquatic animals. High concentrations of ammonia-N are toxic to aquatic life, causing stress, reducing growth rates, and increasing mortality (9, 10). Research has shown that ammonia-N exposure disrupts physiological and metabolic processes in various aquatic species. For example, in rainbow trout (*Oncorhynchus mykiss*), ammonia-N exposure has been shown to disrupt osmoregulation and impair gill function, leading to decreased growth and increased susceptibility to disease (11). Similarly, in zebrafish (*Danio rerio*), chronic exposure to ammonia-N has been linked to altered behavior, impaired reproductive performance, and disruptions in the endocrine system (12). In shrimp, ammonia-N exposure has been shown to affect various biological processes. For instance, in the whiteleg shrimp (*Penaeus vannamei*), high ammonia-N levels have been associated with increased oxidative stress, reduced feed intake, and impaired growth (13). Molecular studies have identified some pathways and genes involved in the ammonia-N stress response, such as those related to oxidative stress, immune response, and energy metabolism (14, 15). Overall, ammonia-N stress exerts wide-ranging impacts on aquatic species, affecting their physiology, metabolism, and survival, highlighting the need for further investigation into its underlying mechanisms. While progress has been made, key gaps remain in understanding the mechanisms behind the integrated responses to ammonia-N exposure. The precise molecular pathways and the comprehensive interaction of various biological processes affected by ammonia-N stress remain largely unknown.

*Penaeus monodon*, is a crucial species in aquaculture due to its high economic value (16, 17). It is often used as a representative species for studying stress responses in crustaceans because of its well-documented physiological and biochemical characteristics (18, 19). Compared to other farmed shrimp species such as *Penaeus vannamei*, *Penaeus monodon* exhibits distinct traits, including a larger body size, higher market value, and greater adaptability to variable environmental conditions, such as salinity and temperature (20, 21). Additionally, *Penaeus monodon* has been reported to show heightened sensitivity to ammonia-N stress, making it a particularly relevant model for this study (22). Several studies have investigated various stress factors affecting *Penaeus monodon*. For example, research on salinity stress has shown significant impacts on the osmoregulatory function and survival rates of this species (23). Studies on thermal stress have highlighted changes in metabolic rates and immune responses (24). Specific to ammonia-N stress, previous studies on *Penaeus monodon* have indicated that high levels of ammonia-N can lead to oxidative stress, immune suppression, and decreased growth rates. For instance, one study demonstrated that exposure to elevated ammonia-N concentrations resulted in significant alterations in the expression of genes related to oxidative stress and immune response (22). Another study found that prolonged ammonia-N exposure adversely affected the metabolic pathways, leading to reduced feed intake and impaired overall health (25). However, the integrated molecular responses to ammonia-N exposure, especially the interactions across biochemical, transcriptomic, and metabolomic pathways, are still not fully elucidated.

This study seeks to address these knowledge gaps by using a multi-omics approach—integrating biochemical, histological, transcriptomic, and metabolomic analyses—to provide a holistic view of the molecular interactions and pathways involved in ammonia-N stress in *Penaeus monodon.* By examining changes in biochemical markers, tissue histology, gene expression, and metabolite profiles at different time points, we aim to reveal the dynamic responses and toxic mechanisms underlying ammonia-N exposure. Insights gained from this study will enhance our understanding of stress response mechanisms in shrimp and contribute to developing strategies to mitigate ammonia-N impacts in aquaculture systems.

## 2 Materials and methods

### 2.1 Animals and ammonia-N exposure experiments

Healthy *Penaeus monodon* shrimp, averaging 7.8 ± 0.4 g, were obtained from the South China Sea Fisheries Research Institute in Shenzhen, China. After a one-week acclimation in laboratory tanks (salinity ~30‰, temperature ~28°C, pH ~7.8), with one-third of the water changed daily, shrimp were fed a commercial diet four times daily.

Preliminary tests were conducted to determine the ammonia-N concentration that could induce significant physiological changes in shrimp while maintaining survival. Based on these tests and previous studies (26, 27), an ammonia-N level of 75 mg/L was chosen, aligning with the 96-hour LC_50_ for this species. To achieve the target concentration, a 10 g/L NH_4_Cl stock solution was used to adjust ammonia-N levels in 500 L tanks. Ammonia-N concentrations were measured every 12 hours following previously established methods (28), and adjustments were made as necessary to maintain consistency. After acclimation, 90 shrimp were randomly divided into three replicates of 30 shrimp in 500 L tanks. NH_4_Cl was added to achieve the target ammonia-N concentration, with water salinity at 30‰, temperature at 28 ± 1°C, pH at 7.5–7.8, and dissolved oxygen above 6.5 mg/L. Samples were collected at 0, 12, and 96 hours, with feeding every six hours at 5% body weight.

### 2.2 Sample collection

Sampling was conducted at 0, 12, and 96 hours to capture the temporal dynamics of ammonia-N stress responses. These time points were chosen based on previous studies and preliminary experiments (22, 25–27). The 0-hour time point represents the baseline physiological and biochemical status before exposure, while 12 and 96 hours reflect the acute and cumulative effects of ammonia-N stress, respectively, including changes linked to the 96-hour LC_50_. At each time point (0, 12, and 96 hours), nine shrimp per replicate were dissected to collect hemolymph and hepatopancreas. Hemolymph was drawn from the ventral sinus using a sterile syringe, while a portion of the hepatopancreas was fixed in 0.4% paraformaldehyde for histology. The rest was frozen in liquid nitrogen and stored at -80°C for RNA extraction and metabolomic analysis.

### 2.3 Biochemical analysis

Serum was obtained by centrifuging hemolymph at 3500 rpm for 10 minutes at 4°C using a high-speed centrifuge (3-18 KS, Sigma, Germany). Blood ammonia and urea nitrogen levels were then measured using assay kits from Nanjing Jiancheng Bioengineering Institute (Nanjing, China).

Hepatopancreas tissues were homogenized in cold 0.9% NaCl solution at a ratio of 1:9 (w/v), and the homogenates were centrifuged according to the kit protocol (4°C, 10 minutes). The activities of Glutamine Synthetase (GS), Superoxide Dismutase (SOD), Xanthine Oxidase (XOD), Adenosine Deaminase (ADA), Caspase-3, Caspase-8, and Aspartate Aminotransferase (AST) in the supernatant were measured using kits from Nanjing Jiancheng Bioengineering Institute.

### 2.4 Hepatopancreas histology

Hepatopancreas samples were randomly collected from shrimp across the three replicates and fixed in 4% paraformaldehyde for 24 hours. The tissues were then rinsed with water and dehydrated using a graded ethanol series. Following dehydration, they were cleared in xylene and embedded in paraffin. Paraffin blocks were sectioned at a thickness of 5 µm using a rotary microtome (Leica RM2125, Germany). The sections were dewaxed and stained with hematoxylin and eosin, then examined and photographed under a microscope (ECLIPSE 200, Nikon, Japan).

### 2.5 Transcriptome sequencing and analysis

To further explore the toxic mechanisms of ammonia-N stress, RNA sequencing was performed on 9 hepatopancreas samples. Total RNA was extracted using TRIzol reagent (Invitrogen, USA), and the concentration and purity of the samples were evaluated with a Nanodrop spectrophotometer. RNA integrity was confirmed through agarose gel electrophoresis. For samples with ≥ 1 µg total RNA, cDNA was synthesized using the NEBNext Ultra II RNA Library Prep Kit (Illumina). The cDNA was then purified with the AMPure XP system and quantified using the Agilent High Sensitivity DNA Assay on a Bioanalyzer 5300 (Agilent).

The sequencing libraries were sequenced on the NovaSeq X Plus platform (Illumina) at Shanghai Majorbio Bio-pharm Biotechnology Co., Ltd. The raw sequencing data were processed with FASTQ (v0.22.0) to obtain high-quality clean reads. Filtered sequences were aligned to the Penaeus monodon reference genome using HISAT2 (v2.1.0), and gene expression was quantified with HTSeq (v0.9.1), then normalized as FPKM (Fragments Per Kilobase of transcript per Million mapped reads). FPKM was chosen for normalization because it is widely adopted in transcriptomic studies for comparing relative expression levels within individual samples. Unlike TPM, which is more suited for cross-sample comparisons, FPKM aligns better with the goals of this study, which focuses on analyzing differential gene expression under specific ammonia-N stress conditions (29–31). Moreover, FPKM is compatible with downstream analysis tools such as DESeq, which was used to identify differentially expressed genes (DEGs) with a threshold of |log2FoldChange| > 1 and P-value < 0.05. All sequences have been submitted to the NCBI SRA (PRJNA1172279).

Gene Ontology (GO) enrichment and Kyoto Encyclopedia of Genes and Genomes (KEGG) pathway analyses were performed using Goatools and Python SciPy libraries. Gene Set Enrichment Analysis (GSEA) (v4.1.0) was applied to all genes, and enriched pathways were visualized through GSEA mapping. Protein-protein interaction networks of DEGs were constructed using STRING (v11.0) with an interaction score > 0.4. Cytoscape (v3.10.1) was used to visualize the networks, revealing interactions among genes under ammonia-N stress. For clarity, the samples were labeled as CG (0 hours), A12h (12 hours), and A96h (96 hours) of ammonia-N exposure.

### 2.6 Metabolomics analysis

Six hepatopancreas samples were collected per time point for non-targeted metabolomics analysis. Metabolites were extracted from all samples following the protocols provided by Metware Biotechnology Co., Ltd. (Wuhan, China) for subsequent Liquid Chromatography-Mass Spectrometry (LC-MS) analysis. Quality control (QC) samples, prepared by mixing equal amounts of metabolites from all samples, were inserted every six samples during analysis to ensure the reproducibility of the procedure. The frequency of QC sample insertion was chosen based on established metabolomic protocols to effectively monitor the stability of the LC-MS system, including retention time, peak intensity, and mass accuracy. This approach ensured the identification and correction of potential technical variation, thereby improving the reliability of the data (32, 33). LC-MS analysis was performed using a UHPLC-QTOF-MS system (AB SCIEX, USA), and the raw data were processed in Progenesis QI software (Waters Co., Milford, MA, USA) for baseline filtering, peak identification, integration, retention time correction, and peak alignment, generating a data matrix with retention time, mass-to-charge ratio, and peak intensity. Missing values were imputed, and response intensities were normalized using the sum normalization method, followed by log10 transformation to produce the final data matrix.

The processed data were analyzed via the Metware Cloud platform (https://cloud.metware.cn) to assess intergroup differences. Partial least squares discriminant analysis (PLS-DA) and orthogonal partial least squares discriminant analysis (OPLS-DA) were carried out using the R package ropls (V1.6.2). Student’s t-test and ANOVA identified significant metabolites, with selection criteria of VIP > 1 and p < 0.05 based on the OPLS-DA model. Metabolite identification was conducted using databases such as HMDB (http://www.hmdb.ca) and METLIN (https://metlin.scripps.edu). HMDB was chosen for its extensive coverage of metabolites, including those found in aquatic organisms, while METLIN provides detailed mass spectrometry data, including accurate mass, retention time, and fragmentation patterns, ensuring high-confidence annotation (34, 35). Metabolic pathways were analyzed using the KEGG database (https://www.kegg.jp). Heatmaps were generated using the default settings of the cloud platform.

### 2.7 Statistical analysis

All data were analyzed using one-way analysis of variance (ANOVA), followed by Tukey’s multiple comparison test in GraphPad Prism (GraphPad Software, La Jolla, CA). Results are presented as mean ± standard error (SE), with statistical significance set at P < 0.05.

## 3 Results

### 3.1 Biochemical analysis

#### 3.1.1 Blood ammonia and urea nitrogen levels

**Figure 1:**
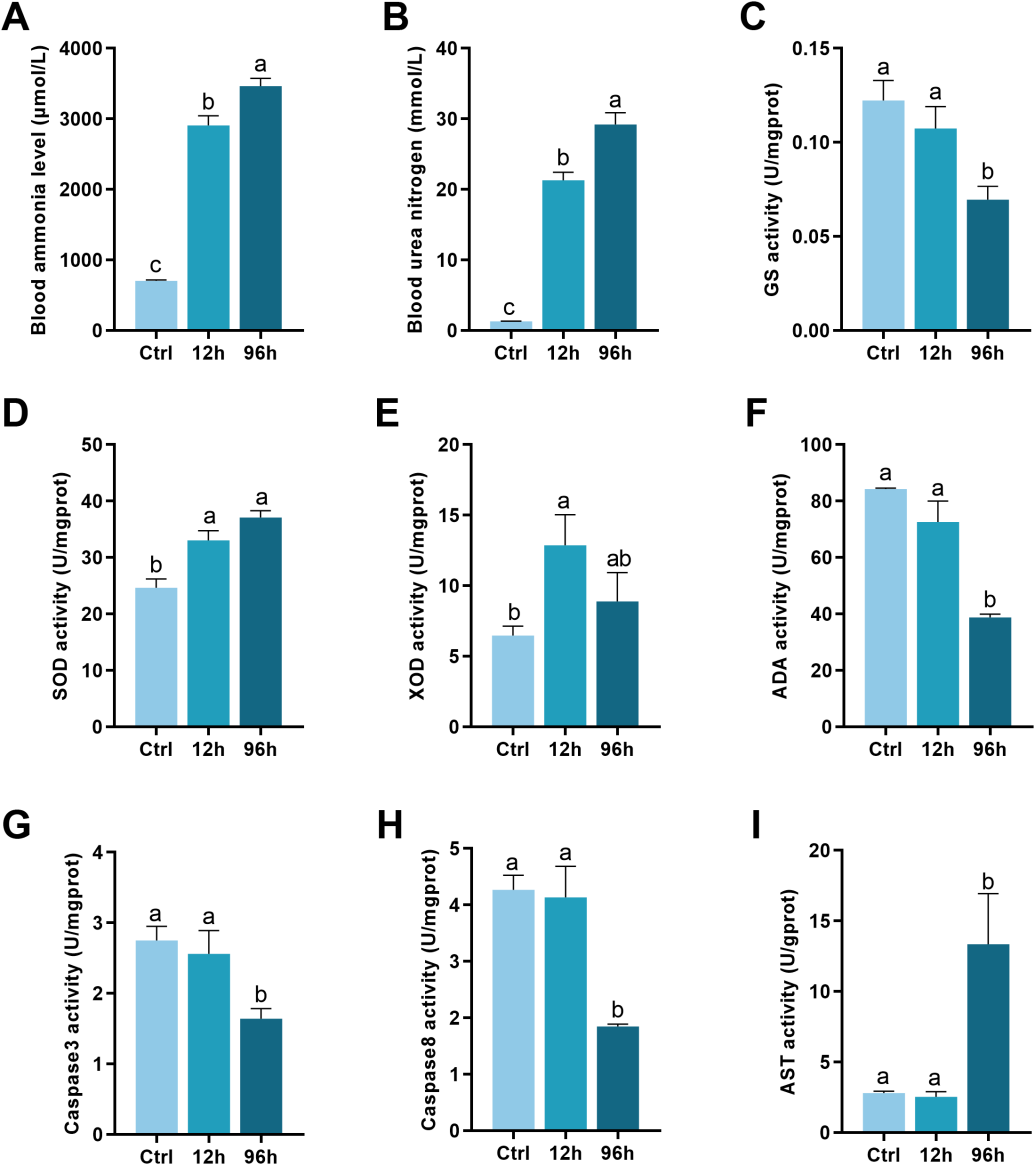
Biochemical Analysis of Shrimp Exposed to Ammonia-N Stress. **(A)** Blood ammonia levels. **(B)** Urea nitrogen levels. **(C)** GS activity. **(D)** SOD activity. **(E)** XOD activity. **(F)** Adenosine deaminase (ADA) activity. **(G)** Caspase-3 activity. **(H)** Caspase-8 activity. **(I)** AST activity. The lowercase letters represent significant differences between groups, as determined by one-way ANOVA. Different letters indicate statistical significance, while the same letter indicates no significant difference.

The blood ammonia level increased significantly with the duration of exposure, with the 96-hour group showing the highest levels (**Figure 1A**, *p* < 0.05). Similar to ammonia levels, urea nitrogen levels were significantly elevated in both the 12-hour and 96-hour exposure groups compared to the control group, with the 96-hour group exhibiting the highest levels (**Figure 1B**, *p* < 0.05).

#### 3.1.2 Enzymatic activities

As shown in **Figure 1C**, the activity of GS decreased significantly after 96 hours of ammonia-N exposure compared to the control and 12-hour groups (*p* < 0.05). SOD activity increased significantly in the 12-hour and 96-hour groups compared to the control group, as presented in **Figure 1D** (*p* < 0.05). **Figure 1E** depicts the XOD activity. XOD activity increased significantly after 12 hours of exposure but showed a slight, non-significant decrease at 96 hours compared to the 12-hour group (*p* < 0.05). ADA activity, as shown in **Figure 1F**, decreased significantly at both 12 hours and 96 hours of ammonia-N exposure compared to the control group (*p* < 0.05).

**Figure 1G** shows that caspase-3 activity remained unchanged between the control and 12-hour groups but decreased significantly after 96 hours of ammonia-N exposure (*p* < 0.05). Similarly, caspase-8 activity (**Figure 1H**) remained constant between the control and 12-hour groups but exhibited a significant reduction in the 96-hour group (*p* < 0.05). AST activity (**Figure 1I**) increased significantly in the 96-hour group compared to both the control and 12-hour groups (*p* < 0.05).

### 3.2 Hepatopancreas histology

**Figure 2:**
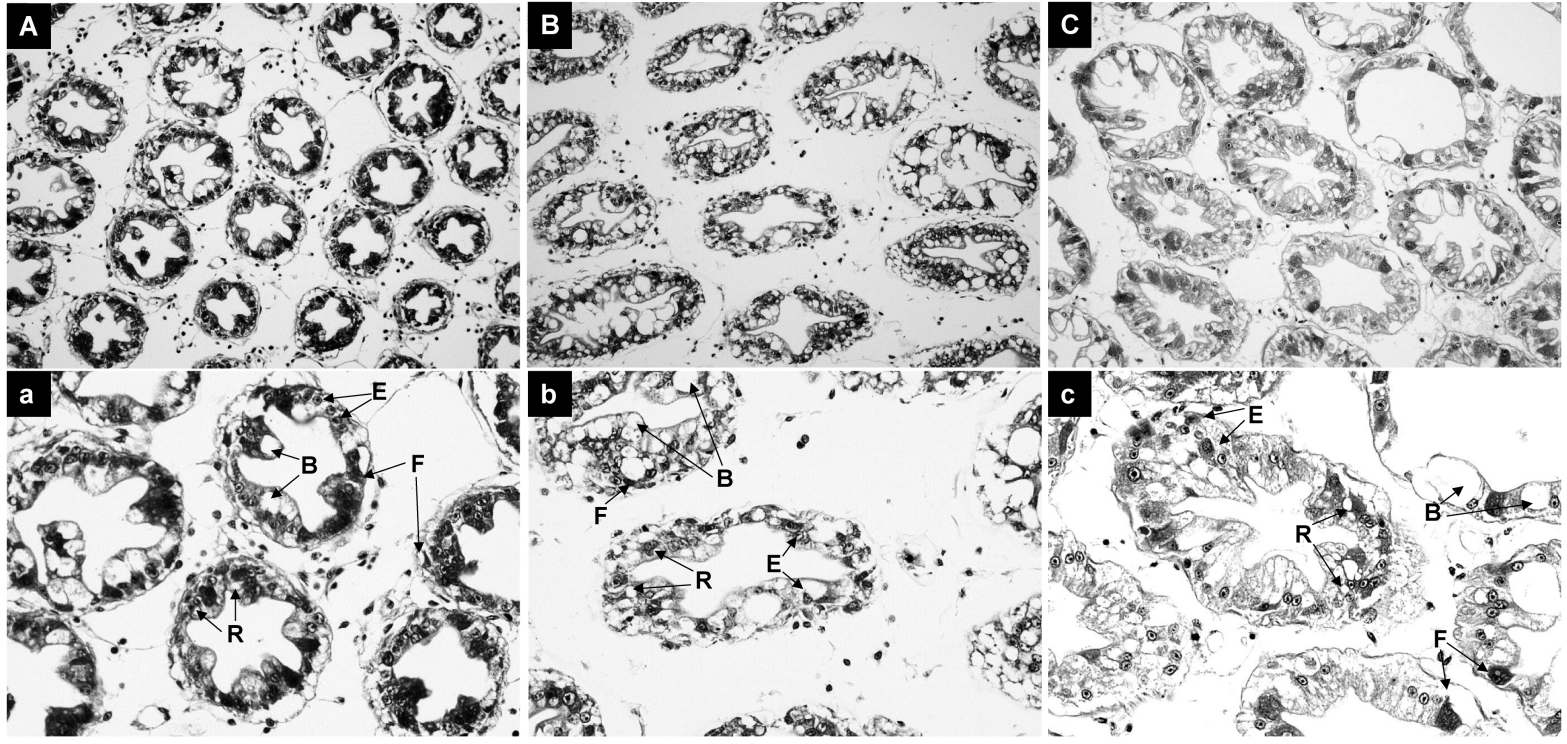
Histological Analysis of Hepatopancreas in Shrimp Exposed to Ammonia-N Stress. **(A)** Control group at 0 hours. **(B)** 12-hour exposure group. **(C)** 96-hour exposure group.

As shown in **Figure 2**, ammonia-N stress caused significant damage to the hepatopancreas of *Penaeus monodon.* Compared to the control group, the severity of hepatopancreas lobule atrophy and irregular luminal deformation increased with longer exposure times (**Figures 2A–C**).

### 3.3 Transcriptome analysis

**Figure 3:**
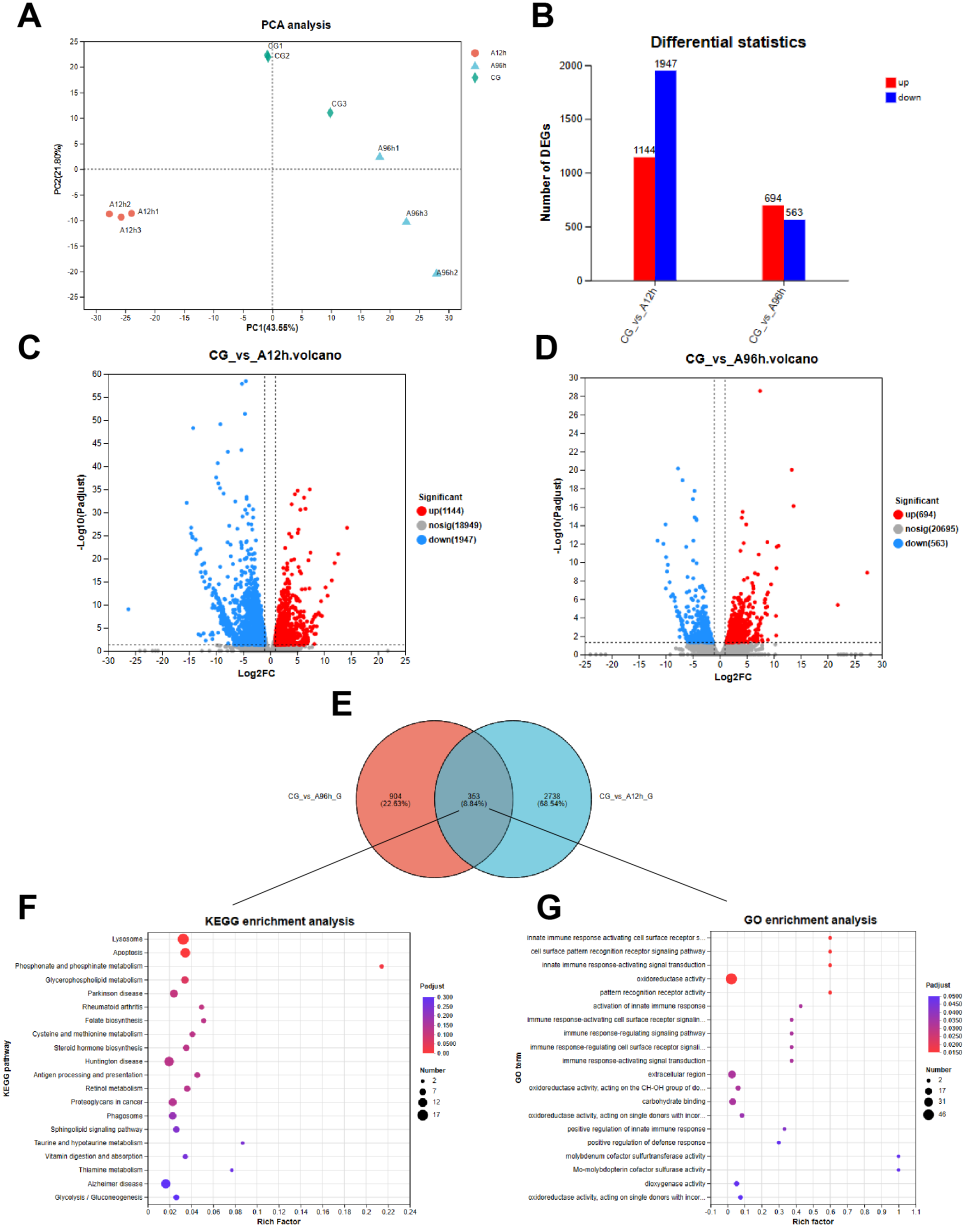
Transcriptome Analysis of Shrimp Exposed to Ammonia-N Stress. **(A)** PCA plot. **(B)** The number of up-regulated and down-regulated genes among all DEGs in each comparison group. **(C)** Venn diagram of DEGs at 12 hours. **(D)** Venn diagram of DEGs at 96 hours. **(E)** Venn diagram of shared DEGs. **(F)** KEGG pathway enrichment. **(G)** GO term enrichment.

Transcriptome analysis was completed for 9 samples, yielding a total of 57.76 Gb of clean data, with each sample producing over 4.57 Gb of clean data. The percentage of Q30 bases was above 92.69% for all samples. The clean reads from each sample were aligned to the genome of *Penaeus monodon*, with alignment rates ranging from 86.12% to 90.4%. Principal Component Analysis (PCA) can reveal the degree of similarity between samples. As shown in **Figure 3A**, samples from the same group clustered closely, indicating good data consistency and high confidence in the transcriptome results (**Figure 3A**).

Compared to the control group, there were 3091 DEGs in the hepatopancreas after 12 hours of ammonia-N stress and 1257 DEGs after 96 hours. Specifically, the 12-hour ammonia-N stress group had 1144 upregulated genes and 1947 downregulated genes, while the 96-hour group had 694 upregulated genes and 563 downregulated genes (**Figures 3B–D**). Notably, there were 353 shared DEGs between the CG *vs* A12h group and the CG *vs* A96h group (**Figure 3E**). Therefore, we further analyzed these shared DEGs to investigate the toxicity mechanisms of ammonia-N stress in shrimp.

#### 3.3.1 KEGG and GO enrichment analysis

KEGG enrichment analysis of the 353 shared differentially expressed genes (DEGs) between the CG *vs* A12h group and the CG *vs* A96h group identified several significantly enriched pathways. The most significantly enriched pathways include lysosome (*P* < 0.001), apoptosis (*P* < 0.001), and phosphonate and phosphinate metabolism (*P* < 0.001). Other notable pathways include glycerophospholipid metabolism, folate biosynthesis, steroid hormone biosynthesis, sphingolipid signaling pathway and glycolysis/gluconeogenesis (**Figure 3F**; **Supplementary Table S1**).

GO enrichment analysis of the 353 shared differentially expressed genes (DEGs) between the CG *vs* A12h group and the CG *vs* A96h group identified several significantly enriched GO terms. The most significantly enriched terms were related to immune response, including “innate immune response activating cell surface receptor signaling pathway”, “cell surface pattern recognition receptor signaling pathway”, and “innate immune response-activating signal transduction” (*P* < 0.001). In addition to immune-related terms, other notable enriched terms included “oxidoreductase activity”, “carbohydrate binding”, and “extracellular region” (**Figure 3G**; **Supplementary Table S2**).

#### 3.3.2 Gene set enrichment analysis

**Figure 4:**
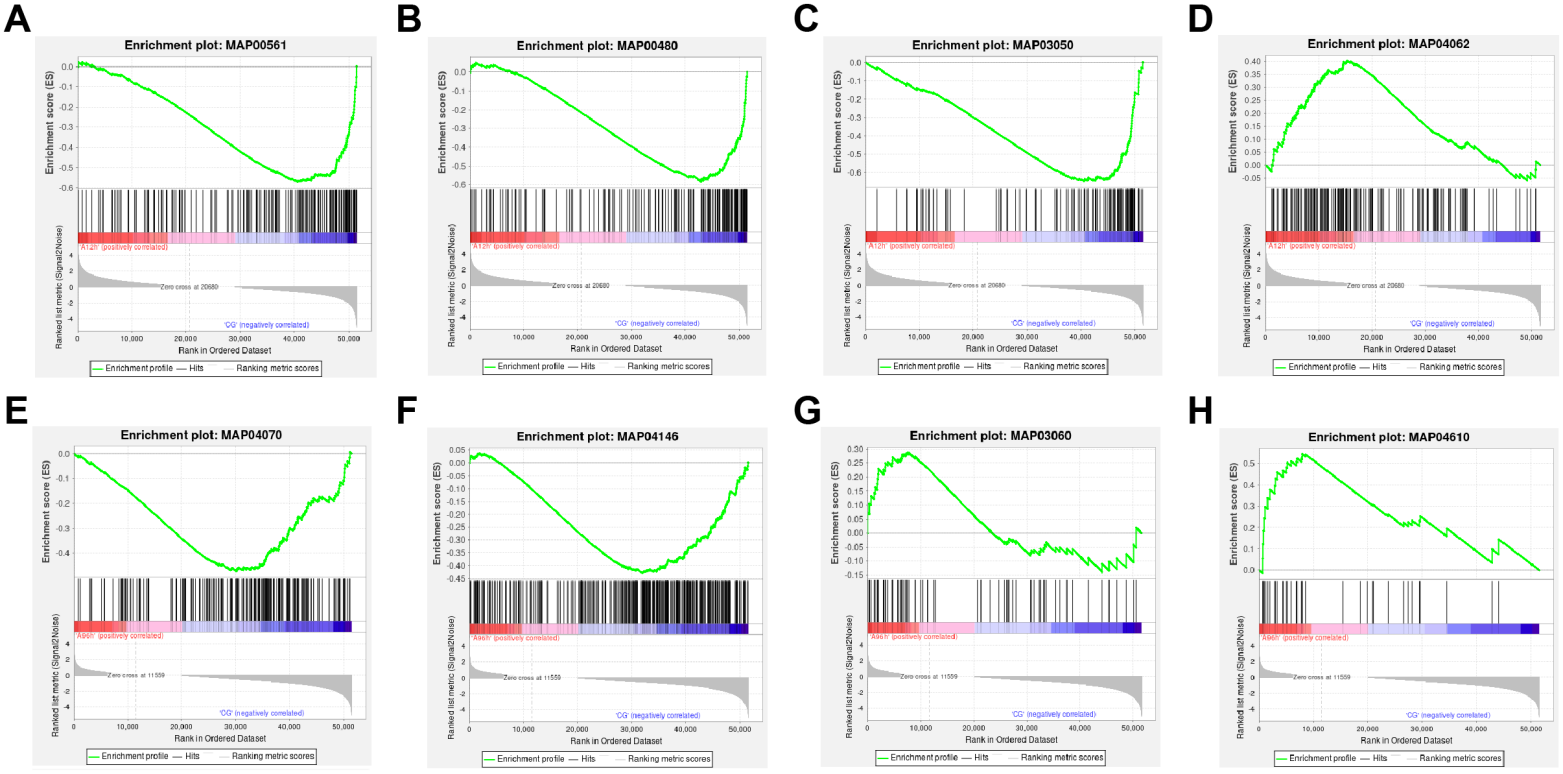
GSEA of Transcriptomic Data. **(A)** Downregulated “Glycerolipid metabolism” pathway at 12 hours. **(B)** Downregulated “Glutathione metabolism” pathway at 12 hours. **(C)** Downregulated “Proteasome” pathway at 12 hours. **(D)** Downregulated “Chemokine signaling” pathway at 12 hours. **(E)** Upregulated “Phosphatidylinositol signaling system” pathway at 96 hours. **(F)** Upregulated “Peroxisome” pathway at 96 hours. **(G)** Downregulated “Protein export” pathway at 96 hours. **(H)** Downregulated “Complement and coagulation cascades” pathway at 96 hours.

The results indicated that the “Glycerolipid metabolism”, “Glutathione metabolism”, and “Proteasome” pathways were significantly downregulated in the 12-hour group, while the “Chemokine signaling” pathway was also significantly downregulated. In contrast, in the 96-hour group, the “Phosphatidylinositol signaling system” and “Peroxisome” pathways were significantly upregulated, whereas the “Protein export” and “Complement and coagulation cascades” pathways were significantly downregulated (|NES| > 1 & *p*-value < 0.05, **Figures 4A–H**).

#### 3.3.3 Protein–protein interaction analysis

**Figure 5:**
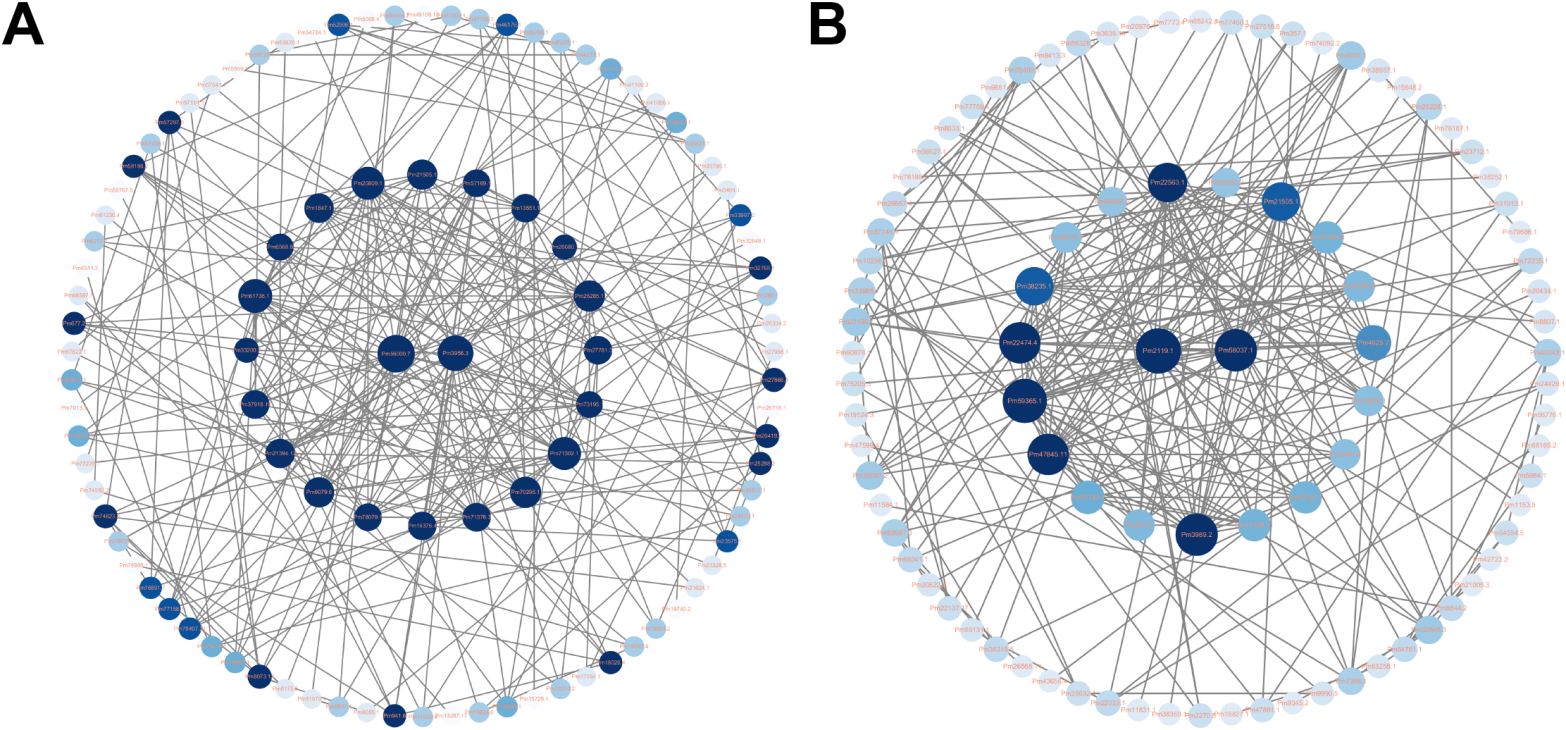
Protein-Protein Interaction (PPI) Networks of DEGs. **(A)** PPI network for 12-hour exposure. **(B)** PPI network for 96-hour exposure.

All DEGs were further mapped to the STRING database for protein-protein interaction (PPI) analysis. DEGs with interaction scores greater than 0.4 were selected for further analysis. The results showed that the CG *vs* A12h group contained 100 proteins, while the CG *vs* A96h group contained 91 proteins (**Figures 5A, B**). In the CG *vs* A12h group, the small subunit ribosomal protein S9e (*RP-S9e*) and citrate synthase (*CS*) were identified as the most highly connected genes. These genes are involved in the “Ribosome” and “Citrate cycle (TCA cycle)” pathways, respectively. In the CG *vs* A96h group, the large subunit ribosomal protein L23e (*RP-L23e*) and phosphoribosylamine–glycine ligase (*GART*) were the most highly connected genes, associated with the “Ribosome” and “Purine metabolism” pathways, respectively (**Supplementary Table S3**).

### 3.4 Metabolomics analysis

**Figure 6:**
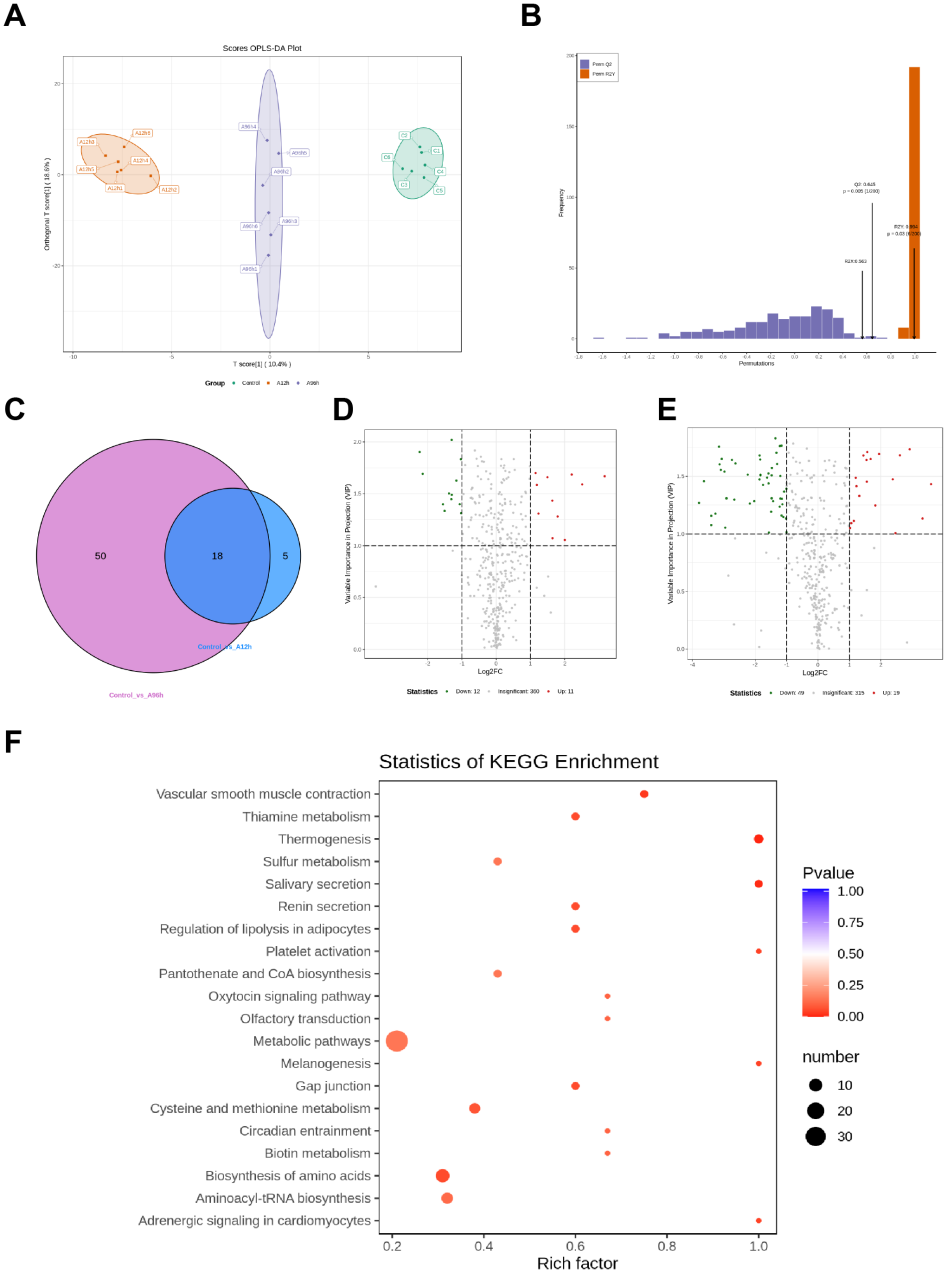
Metabolomic Analysis of Shrimp Exposed to Ammonia-N Stress. **(A)** OPLS-DA score plot. **(B)** Permutation test results. **(C)** Venn diagram of shared differential metabolites. **(D)** Heatmap of upregulated metabolites at 12 and 96 hours. **(E)** Heatmap of downregulated metabolites at 12 and 96 hours. **(F)** KEGG pathway enrichment.

The OPLS-DA score plot shows clear separation among the control, 12-hour ammonia-N stress (A12h), and 96-hour ammonia-N stress (A96h) groups, indicating distinct metabolic profiles for each condition. Permutation tests confirm the model’s robustness and predictive ability, with an R2Y value of 0.994 (p = 0.03) and a Q2 value of 0.645 (*p* = 0.005), demonstrating reliable differentiation between the groups (**Figures 6A, B**). Compared to the control group, the 12-hour ammonia-N stress group (A12h) exhibited 23 significantly different metabolites, while the 96-hour ammonia-N stress group (A96h) had 68 significantly different metabolites. Specifically, in the CG *vs* A12h group, there were 11 upregulated metabolites and 12 downregulated metabolites. In the CG *vs* A96h group, there were 19 upregulated metabolites and 49 downregulated metabolites (**Figures 6D, E**). Notably, there were 18 shared metabolites between the CG *vs* A12h group and the CG *vs* A96h group (**Figure 6C**). These shared metabolites were subjected to further investigation (**Figure 6C**).

#### 3.4.1 KEGG enrichment analysis

KEGG enrichment analysis of the 18 shared differential metabolites between the CG *vs* A12h group and the CG *vs* A96h group identified several significantly enriched pathways. Key pathways include metabolic pathways, thiamine metabolism, sulfur metabolism, regulation of lipolysis in adipocytes, platelet activation, pantothenate and CoA biosynthesis, and cysteine and methionine metabolism (**Figure 6F**).

#### 3.4.2 Time-dependent upregulated metabolites

**Figure 7:**
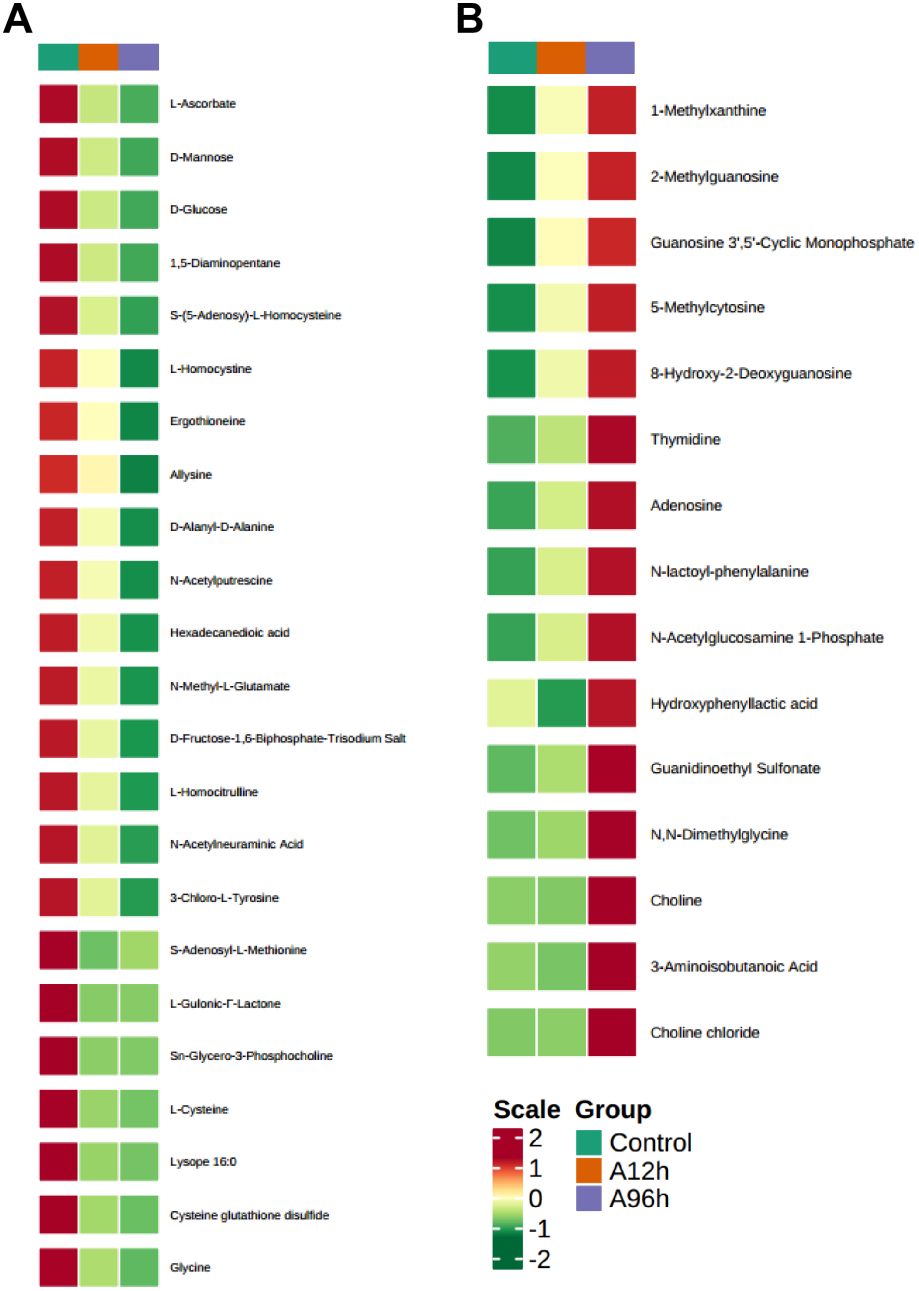
Heatmaps of Time-Dependent Metabolite Changes. **(A)** Heatmap of upregulated metabolites over time. **(B)** Heatmap of downregulated metabolites over time.

To further investigate the effects of ammonia-N stress on shrimp, we analyzed the metabolites that were upregulated over time using a heatmap (**Figure 7A**). The analysis identified several key metabolites with significant time-dependent upregulation. Notably, 1-Methylxanthine increased 1.702-fold at 12 hours and 2.489-fold at 96 hours (*p* = 0.064 at 12h, *p* = 0.044 at 96h). Similarly, 2-Methylguanosine increased 4.011-fold at 12 hours and 7.146-fold at 96 hours (*p* = 0.016 at 12h, *p* = 0.133 at 96h), while Guanosine 3’,5’-Cyclic Monophosphate showed a 5.702-fold increase at 12 hours and a 10.048-fold increase at 96 hours (*p* = 0.026 at 12h, *p* = 0.056 at 96h). 5-Methylcytosine and 8-Hydroxy-2-Deoxyguanosine exhibited significant increases, with 5-Methylcytosine increasing 1.864-fold at 12 hours and 2.935-fold at 96 hours (*p* = 0.118 at 12h, *p* = 0.009 at 96h) and 8-Hydroxy-2-Deoxyguanosine increasing 1.827-fold at 12 hours and 2.909-fold at 96 hours (*p* = 0.045 at 12h, *p* < 0.001 at 96h). Thymidine increased 3.467-fold at 12 hours and 12.099-fold at 96 hours (*p* = 0.039 at 12h, *p* = 0.137 at 96h), and Adenosine increased 1.312-fold at 12 hours and 2.031-fold at 96 hours (*p* = 0.359 at 12h, *p* = 0.124 at 96h). Additionally, N-lactoyl-phenylalanine showed a 3.115-fold increase at 12 hours and a 7.562-fold increase at 96 hours (*p* = 0.014 at 12h, *p* = 0.003 at 96h) (**Supplementary Table S4**).

#### 3.4.3 Time-dependent downregulated metabolites

To further investigate the effects of ammonia-N stress on shrimp, we analyzed the metabolites that were consistently downregulated over time using a heatmap (**Figure 7B**). The analysis identified several key metabolites that showed significant time-dependent downregulation. Notably, L-Ascorbate decreased to 0.416-fold at 12 hours and 0.222-fold at 96 hours (*p* = 0.011 at 12h, *p* = 0.003 at 96h). D-Mannose and D-Glucose showed similar trends, with D-Mannose decreasing to 0.342-fold at 12 hours and 0.081-fold at 96 hours (*p* = 0.054 at 12h, *p* = 0.016 at 96h) and D-Glucose decreasing to 0.352-fold at 12 hours and 0.104-fold at 96 hours (*p* = 0.051 at 12h, *p* = 0.016 at 96h). Other significantly downregulated metabolites include 1,5-Diaminopentane, which decreased to 0.479-fold at 12 hours and 0.281-fold at 96 hours (*p* = 0.041 at 12h, *p* = 0.011 at 96h), and S-(5-Adenosy)-L-Homocysteine, which decreased to 0.519-fold at 12 hours and 0.281-fold at 96 hours (*p* = 0.03 at 12h, *p* = 0.005 at 96h). L-Homocystine and Ergothioneine also showed significant reductions, with L-Homocystine decreasing to 0.723-fold at 12 hours and 0.456-fold at 96 hours (*p* = 0.204 at 12h, *p* = 0.019 at 96h) and Ergothioneine decreasing to 0.676-fold at 12 hours and 0.335-fold at 96 hours (*p* = 0.199 at 12h, *p* = 0.006 at 96h). Furthermore, metabolites such as Allysine, D-Alanyl-D-Alanine, N-Acetylputrescine, and Hexadecanedioic acid exhibited significant downregulation over time. Allysine decreased to 0.619-fold at 12 hours and 0.158-fold at 96 hours (*p* = 0.417 at 12h, *p* = 0.01 at 96h), while D-Alanyl-D-Alanine decreased to 0.692-fold at 12 hours and 0.431-fold at 96 hours (*p* = 0.007 at 12h, *p* < 0.001 at 96h) (**Supplementary Table S5**).

## 4 Discussion

Ammonia stress is one of the most common harmful toxins in aquaculture environments, yet our understanding of the molecular mechanisms underlying shrimp tolerance to ammonia stress remains quite limited. In this study, we systematically investigated the transcriptomic changes in black tiger shrimp (*Penaeus monodon*) under ammonia stress through the measurement of key biochemical indicators, histological analysis, RNA-seq analysis, and metabolomic analysis. Our findings provide new insights into the physiological and molecular mechanisms of ammonia stress on shrimp. Compared to other stressors in aquaculture, such as salinity and temperature fluctuations, ammonia stress has a direct and immediate toxic effect due to the accumulation of unionized ammonia, which readily disrupts metabolic processes, damages tissues, and compromises immune function. These unique characteristics make ammonia stress a critical focus for aquaculture research.

Ammonia-N stress leads to severe tissue damage. The hepatopancreas in crustaceans, including *Penaeus monodon*, plays a crucial role in digestion, absorption, and storage of nutrients, as well as detoxification processes (36, 37). In this study, the severity of hepatopancreas lobule atrophy and irregular luminal deformation increased with longer exposure times. These histological changes align with findings from studies on other aquatic species, such as whiteleg shrimp (*Penaeus vannamei*) and zebrafish (*Danio rerio*), where ammonia exposure similarly resulted in tissue damage, including cellular swelling, necrosis, and luminal deformation (38, 39). The consistency of these findings across different species suggests a common pathological response to ammonia toxicity. The observed tissue damage likely results from the cumulative toxic effects of ammonia, including oxidative stress and disruption of cellular homeostasis (9, 40).

Ammonia-N stress disrupts nitrogen metabolism, induces oxidative stress, and impairs detoxification mechanisms. The significant increase in blood ammonia levels with prolonged exposure, particularly in the 96-hour group, suggests that ammonia-N stress can overwhelm the shrimp’s natural detoxification processes, leading to systemic toxicity. Elevated ammonia levels in the blood can disrupt osmoregulation and impair gill function, which are critical for maintaining homeostasis in aquatic organisms (41). This disruption is likely to result in decreased growth and increased susceptibility to disease, as observed in other species like rainbow trout (*Oncorhynchus mykiss*) (42). The increase in urea nitrogen levels indicates heightened protein catabolism and impaired excretion processes, further stressing the organism’s metabolic systems (43). The significant decrease in GS activity points to a compromised ability to detoxify ammonia through its conversion to glutamine. GS is essential for mitigating ammonia toxicity by incorporating ammonia into amino acids, and its reduced activity suggests that shrimp under prolonged ammonia exposure may suffer from ammonia accumulation, leading to cellular damage and metabolic dysfunction (44). This aligns with findings in zebrafish (*Danio rerio*), where chronic ammonia exposure disrupts nitrogen metabolism (45). The increase in SOD activity reflects an adaptive response to oxidative stress induced by ammonia-N exposure. SOD is a crucial antioxidant enzyme that mitigates the damaging effects of ROS (46). Elevated SOD activity suggests that shrimp are experiencing oxidative stress and are attempting to counteract the resultant cellular damage. This adaptive response is consistent with observations in whiteleg shrimp (*Penaeus vannamei*) and indicates a conserved mechanism across species to manage oxidative stress (38). The changes in XOD and ADA activities highlight disruptions in purine metabolism, which can lead to the accumulation of toxic metabolites and further oxidative stress. XOD generates ROS during purine metabolism, and its fluctuating activity suggests an initial stress response followed by potential substrate depletion or adaptive regulation (47). Reduced ADA activity indicates a backlog in purine catabolism, which could contribute to metabolic imbalances and stress (48). The decreased activities of caspase-3 and caspase-8 after 96 hours suggest an inhibition of apoptotic pathways (49). Apoptosis is a controlled mechanism for eliminating damaged cells (50), and its inhibition could indicate that ammonia-N stress is causing cellular damage without triggering adequate cell death responses. This could result in the accumulation of damaged cells, exacerbating tissue dysfunction and impairing overall health. Similar effects have been observed in fish under prolonged stress conditions, where apoptosis pathways are altered (51). Elevated AST activity serves as a marker of tissue damage and increased protein turnover (52). The significant rise in AST activity indicates that ammonia-N exposure causes cellular damage, likely due to the combined effects of oxidative stress and disrupted metabolic processes. Increased AST activity suggests heightened amino acid catabolism, which may be a compensatory response to meet the energy demands under stress conditions (53).

Transcriptome analysis indicates that ammonia-N stress significantly impacts shrimp health by inducing apoptosis, disrupting metabolic processes, and activating immune responses. The identification of a substantial number of DEGs at both 12 and 96 hours reflects a robust and dynamic response to ammonia exposure. KEGG and GO enrichment analyses revealed that critical pathways involved in apoptosis, lysosomal function, and phosphonate metabolism are significantly affected. The enrichment of apoptosis-related pathways suggests that ammonia-N stress triggers cell death processes, which aligns with findings in other aquatic species (38). For instance, similar activation of apoptotic pathways in response to ammonia exposure has been documented in zebrafish and rainbow trout, indicating a conserved mechanism of ammonia-induced cellular stress across different species (54, 55). The involvement of lysosomal pathways suggests that autophagy plays a role in the response to ammonia-N stress (56). Autophagy is a conserved cellular degradation process that maintains cellular homeostasis by removing damaged organelles and misfolded proteins, especially under environmental stress conditions (57, 58). Evidence from other species highlights its importance during ammonia exposure. For example, studies in shrimp such as Pacific whiteleg shrimp (*Litopenaeus vannamei*) have shown that sub-lethal ammonia stress induces upregulation of autophagy-related genes, including ATG3, ATG4, and ATG12, alongside tissue damage in the hepatopancreas (59). These findings suggest that autophagy functions as a protective mechanism to mitigate ammonia-induced cellular damage. In *Penaeus monodon*, the activation of lysosomal pathways observed in our study suggests that autophagy plays a protective role against ammonia-induced damage. By degrading damaged cellular components, autophagy helps mitigate oxidative stress and supports cellular recovery. This mechanism aligns with responses observed in other aquatic species, highlighting the conserved role of autophagy in managing environmental stressors. The immune-related GO terms highlight the activation of the immune response as a significant aspect of the shrimp’s reaction to ammonia-N stress. This is consistent with observations in other aquatic organisms, where pollutants and environmental stressors often lead to immune system activation as a defensive response (60, 61). GSEA provided further insights (62), showing downregulation of crucial metabolic pathways such as glycerolipid metabolism and glutathione metabolism at the early stages of exposure. This downregulation may reflect an initial metabolic slowdown as the organism attempts to conserve energy and resources in response to stress (63, 64). In contrast, the later stages showed upregulation in pathways related to phosphatidylinositol signaling and peroxisome function, indicating a shift towards recovery and cellular repair mechanisms (65, 66). PPI analysis identified highly connected hub proteins involved in ribosomal function and purine metabolism. The centrality of ribosomal proteins underscores the importance of protein synthesis in the stress response, as ribosome biogenesis is critical for cell survival and function under stress conditions (67). The identification of citrate synthase and phosphoribosylamine-glycine ligase suggests that these genes may play crucial roles in the response to ammonia-N stress, given their pivotal functions in energy production and nucleotide synthesis (68, 69), respectively.

Metabolomic analysis reveals that ammonia-N stress significantly disrupts shrimp metabolism, causing changes in nucleotide turnover, antioxidant defenses, and fundamental metabolic pathways. Identifying 23 significantly different metabolites in the A12h group and 68 in the A96h group suggests that the metabolic impact of ammonia-N stress intensifies over time. The 18 shared differential metabolites between the two stress groups provide a consistent metabolic signature of ammonia-N exposure, highlighting critical biochemical pathways affected by this stressor. KEGG enrichment analysis of these shared metabolites identified significant pathways involved in metabolic regulation, such as thiamine metabolism, sulfur metabolism, regulation of lipolysis in adipocytes, and cysteine and methionine metabolism. These pathways are crucial for maintaining cellular homeostasis and energy production (70–73), indicating that ammonia-N stress disrupts fundamental metabolic processes. Time-dependent changes in specific metabolites further elucidate the biochemical impact of ammonia-N stress. The upregulation of metabolites such as 1-Methylxanthine, 2-Methylguanosine, and Guanosine 3’,5’-Cyclic Monophosphate over time indicates enhanced nucleotide metabolism and possibly a stress-induced increase in DNA and RNA turnover (74–76). This response could be a protective mechanism to repair ammonia-induced genetic damage. Conversely, the downregulation of metabolites such as L-Ascorbate, D-Mannose, and D-Glucose suggests a depletion of essential nutrients and antioxidants over prolonged exposure. The significant reduction in L-Ascorbate, a critical antioxidant, indicates increased oxidative stress (77), corroborating the observed increase in oxidative stress markers like SOD activity. The decrease in metabolites like 1,5-Diaminopentane and S-(5-Adenosy)-L-Homocysteine, involved in amino acid and sulfur metabolism, further highlights disruptions in nitrogen and sulfur metabolic pathways (78, 79). Such disruptions can impair protein synthesis and cellular detoxification processes, exacerbating the physiological stress on the organism.

In summary, this study found that ammonia-N stress severely impacts shrimp health by causing tissue damage in the hepatopancreas, disrupting nitrogen metabolism, inducing oxidative stress, and impairing detoxification mechanisms. Transcriptomic analysis revealed significant effects on apoptosis, metabolic processes, and immune responses, while metabolomic analysis showed disruptions in nucleotide turnover, antioxidant defenses, and key metabolic pathways. These findings highlight the critical effects of ammonia-N stress on shrimp and underscore the importance of further research to mitigate these impacts in aquaculture systems.

## 5 Conclusions

This study demonstrates that ammonia-N stress significantly impacts shrimp health by causing hepatopancreatic damage and altering immune responses, metabolic pathways, and gene expression profiles. Although the initial effects are relatively minor, prolonged exposure results in severe histological damage, particularly atrophy and deformation of the hepatopancreas. By evaluating key markers of oxidative stress, nitrogen metabolism, immune response, and overall health, we gained critical insights into the underlying mechanisms of ammonia-N toxicity. Transcriptomic analysis revealed the involvement of apoptosis, immune activation, and key metabolic pathways in the hepatopancreas. Integrating transcriptomic and metabolomic data further highlighted disruptions in immune-related signaling, nucleotide turnover, antioxidant defenses, and fundamental metabolic processes, indicating a multifaceted stress response. These findings suggest that shrimp may employ adaptive immune and metabolic strategies under ammonia-N stress, though further research is needed to explore deeper molecular mechanisms. Future studies should focus on clarifying immune-related pathways and developing interventions to enhance shrimp resilience to environmental and immunotoxic stressors. This research provides valuable molecular-level data on crustacean immune and physiological responses to environmental stress and lays the foundation for assessing the ecological risks of ammonia-N while identifying biomarkers for mitigating its adverse effects in aquaculture systems.

## Data Availability

The datasets presented in this study can be found in online repositories. The names of the repository/repositories and accession number(s) can be found below: https://www.ncbi.nlm.nih.gov/, PRJNA1172279.
