# Supplementary material for: Immune and physiological responses in Penaeus monodon to ammonia-N stress: a multi-omics approach
Source: Front Immunol. 2024 Dec 10;15:1510887. doi: 10.3389/fimmu.2024.1510887 (PMC11666502; doi:10.3389/fimmu.2024.1510887)
Supplement: Supplementary file 1 [file DataSheet1.docx]

**Supplementary data for “Immune and Physiological Responses in Penaeus monodon to Ammonia-N Stress: A Multi-Omics Approach”**

Zhi Luo ^1,3^, Falin Zhou ^1,2^, Song Jiang ^1,2^, Jianhua Huang ^1,3,4^, Lishi Yang ^1,2^, Qibin Yang ^1,2,4^, Jianzhi Shi ^1,2^, Erchao Li ^3^, Zhenhua Ma ^1,2^, Yundong Li ^1,2,3,4*^

1 South China Sea Fisheries Research Institute, Chinese Academy of Fishery Sciences, Key Laboratory of South China Sea Fishery Resources Exploitation and Utilization, Ministry of Agriculture and Rural Affairs, Guangzhou 510300, China

2 Key Laboratory of Efficient Utilization and Processing of Marine Fishery Resources of Hainan Province, Sanya Tropical Fisheries Research Institute, Sanya 572018, China

3 Laboratory of Aquaculture Nutrition and Environmental Health, School of Life Sciences, East China Normal University, 500 Dongchuan Road, Shanghai 200241, China

4 Shenzhen Base of South China Sea Fisheries Research Institute, Chinese Academy of Fishery Sciences, Shenzhen 518108, China

Corresponding authors:

*Yundong Li: liyd2019@163.com

**Supplementary Table**

| Pathway id | Description | Pvalue | First Category | Second Category |
| --- | --- | --- | --- | --- |
| map04142 | Lysosome | 0.000 | Cellular Processes | Transport and catabolism |
| map04210 | Apoptosis | 0.000 | Cellular Processes | Cell growth and death |
| map00440 | Phosphonate and phosphinate metabolism | 0.000 | Metabolism | Metabolism of other amino acids |
| map00564 | Glycerophospholipid metabolism | 0.001 | Metabolism | Lipid metabolism |
| map05012 | Parkinson disease | 0.006 | Human Diseases | Neurodegenerative disease |
| map05323 | Rheumatoid arthritis | 0.005 | Human Diseases | Immune disease |
| map00790 | Folate biosynthesis | 0.005 | Metabolism | Metabolism of cofactors and vitamins |
| map00270 | Cysteine and methionine metabolism | 0.004 | Metabolism | Amino acid metabolism |
| map00140 | Steroid hormone biosynthesis | 0.004 | Metabolism | Lipid metabolism |
| map05016 | Huntington disease | 0.005 | Human Diseases | Neurodegenerative disease |
| map04612 | Antigen processing and presentation | 0.003 | Organismal Systems | Immune system |
| map00830 | Retinol metabolism | 0.003 | Metabolism | Metabolism of cofactors and vitamins |
| map05205 | Proteoglycans in cancer | 0.008 | Human Diseases | Cancer: overview |
| map04145 | Phagosome | 0.012 | Cellular Processes | Transport and catabolism |
| map04071 | Sphingolipid signaling pathway | 0.015 | Environmental Information Processing | Signal transduction |
| map00430 | Taurine and hypotaurine metabolism | 0.017 | Metabolism | Metabolism of other amino acids |
| map04977 | Vitamin digestion and absorption | 0.019 | Organismal Systems | Digestive system |
| map00730 | Thiamine metabolism | 0.021 | Metabolism | Metabolism of cofactors and vitamins |
| map05010 | Alzheimer disease | 0.025 | Human Diseases | Neurodegenerative disease |
| map00010 | Glycolysis / Gluconeogenesis | 0.026 | Metabolism | Carbohydrate metabolism |

Table S1 Significantly Enriched Pathways Identified in KEGG Enrichment Analysis of Shared Differential Metabolites.

Table S2 Significantly Enriched GO Terms Identified in GO Enrichment Analysis of Shared DEGs.

| GO ID | Term Type | Description | Pvalue |
| --- | --- | --- | --- |
| GO:0002220 | BP | innate immune response activating cell surface receptor signaling pathway | 0.000 |
| GO:0002752 | BP | cell surface pattern recognition receptor signaling pathway | 0.000 |
| GO:0002758 | BP | innate immune response-activating signal transduction | 0.000 |
| GO:0016491 | MF | oxidoreductase activity | 0.000 |
| GO:0038187 | MF | pattern recognition receptor activity | 0.000 |
| GO:0002218 | BP | activation of innate immune response | 0.000 |
| GO:0002429 | BP | immune response-activating cell surface receptor signaling pathway | 0.000 |
| GO:0002764 | BP | immune response-regulating signaling pathway | 0.000 |
| GO:0002768 | BP | immune response-regulating cell surface receptor signaling pathway | 0.000 |
| GO:0002757 | BP | immune response-activating signal transduction | 0.000 |
| GO:0005576 | CC | extracellular region | 0.000 |
| GO:0016616 | MF | oxidoreductase activity, acting on the CH-OH group of donors, NAD or NADP as acceptor | 0.000 |
| GO:0030246 | MF | carbohydrate binding | 0.000 |
| GO:0016702 | MF | oxidoreductase activity, acting on single donors with incorporation of molecular oxygen, incorporation of two atoms of oxygen | 0.000 |
| GO:0045089 | BP | positive regulation of innate immune response | 0.000 |
| GO:0031349 | BP | positive regulation of defense response | 0.000 |
| GO:0102867 | MF | molybdenum cofactor sulfurtransferase activity | 0.000 |
| GO:0008265 | MF | Mo-molybdopterin cofactor sulfurase activity | 0.000 |
| GO:0051213 | MF | dioxygenase activity | 0.000 |
| GO:0016701 | MF | oxidoreductase activity, acting on single donors with incorporation of molecular oxygen | 0.000 |

Table S3 The annotation of genes with the highest connectivity in the protein interaction network of each group.

| Group | Node name | Node degree | NR description (KO name) | KEGG pathway | Pathway Definition |
| --- | --- | --- | --- | --- | --- |
| Ctrl vs 12h | *Pm56000.7* | 22 | small subunit ribosomal protein S9e (*RP-S9e*) | map03010 | Ribosome |
|  | *Pm3956.3* | 20 | citrate synthase (*CS*) | map00020 | Citrate cycle (TCA cycle) |
| Ctrl vs 96h | *Pm2119.1* | 24 | large subunit ribosomal protein L23e (*RP-L23e*) | map03010 | Ribosome |
|  | *Pm58037.1* | 21 | phosphoribosylamine--glycine ligase (*GART*) | map00230 | Purine metabolism |

Table S4 Metabolites Downregulated Over Time Due to Ammonia-N Exposure.

| Index | Compounds | Fold Change 12h | Fold Change 96h | *p*-value 12h | *p*-value 96h |
| --- | --- | --- | --- | --- | --- |
| MEDN242 | L-Ascorbate | 0.416 | 0.222 | 0.011 | 0.003 |
| MEDN221 | D-Mannose | 0.342 | 0.081 | 0.054 | 0.016 |
| MEDN220 | D-Glucose | 0.352 | 0.104 | 0.051 | 0.016 |
| MEDP126 | 1,5-Diaminopentane | 0.479 | 0.281 | 0.041 | 0.011 |
| MEDN068 | S-(5-Adenosy)-L-Homocysteine | 0.519 | 0.281 | 0.03 | 0.005 |
| MEDP056 | L-Homocystine | 0.723 | 0.456 | 0.204 | 0.019 |
| MEDP437 | Ergothioneine | 0.676 | 0.335 | 0.199 | 0.006 |
| MEDN487 | Allysine | 0.619 | 0.158 | 0.417 | 0.01 |
| MEDN036 | D-Alanyl-D-Alanine | 0.692 | 0.431 | 0.007 | 0 |
| MEDP069 | N-Acetylputrescine | 0.508 | 0.095 | 0.175 | 0.031 |
| MEDN658 | Hexadecanedioic acid | 0.685 | 0.448 | 0.251 | 0.052 |
| MEDP878 | N-Methyl-L-Glutamate | 0.575 | 0.279 | 0.127 | 0.023 |
| MEDN485 | D-Fructose-1,6-Biphosphate-Trisodium Salt | 0.564 | 0.273 | 0.166 | 0.036 |
| MEDP055 | L-Homocitrulline | 0.607 | 0.36 | 0.05 | 0.004 |
| MEDN058 | N-Acetylneuraminic Acid | 0.408 | 0.072 | 0.044 | 0.005 |
| MEDP031 | 3-Chloro-L-Tyrosine | 0.594 | 0.36 | 0.103 | 0.016 |
| MEDP080 | S-Adenosyl-L-Methionine | 0.404 | 0.465 | 0.058 | 0.08 |
| MEDN240 | L-Gulonic-γ-Lactone | 0.407 | 0.406 | 0 | 0 |
| MEDP442 | Sn-Glycero-3-Phosphocholine | 0.385 | 0.371 | 0.077 | 0.074 |
| MEDP052 | L-Cysteine | 0.227 | 0.162 | 0.007 | 0.005 |
| MEDN366 | Lysope 16:0 | 0.49 | 0.451 | 0 | 0.04 |
| MEDN822 | Cysteine glutathione disulfide | 0.213 | 0.113 | 0.011 | 0.007 |
| MEDP006 | Glycine | 0.534 | 0.447 | 0.003 | 0.001 |

Table S5 Metabolites Upregulated Over Time Due to Ammonia-N Exposure.

| Index | Compounds | Fold Change 12h | Fold Change 96h | *p*-value 12h | *p*-value 96h |
| --- | --- | --- | --- | --- | --- |
| MEDP148 | 1-Methylxanthine | 1.702 | 2.489 | 0.064 | 0.044 |
| MEDN568 | 2-Methylguanosine | 4.011 | 7.146 | 0.016 | 0.133 |
| MEDN161 | Guanosine 3',5'-Cyclic Monophosphate | 5.702 | 10.048 | 0.026 | 0.056 |
| MEDP155 | 5-Methylcytosine | 1.864 | 2.935 | 0.118 | 0.009 |
| MEDP382 | 8-Hydroxy-2-Deoxyguanosine | 1.827 | 2.909 | 0.045 | 0 |
| MEDN168 | Thymidine | 3.467 | 12.099 | 0.039 | 0.137 |
| MEDP160 | Adenosine | 1.312 | 2.031 | 0.359 | 0.124 |
| MEDN579 | N-lactoyl-phenylalanine | 3.115 | 7.562 | 0.014 | 0.003 |
| MEDN506 | N-Acetylglucosamine 1-Phosphate | 1.413 | 2.296 | 0.056 | 0.014 |
| MEDN555 | Hydroxyphenyllactic acid | 0.494 | 1.872 | 0.03 | 0.066 |
| MEDN316 | Guanidinoethyl Sulfonate | 1.808 | 6.099 | 0.007 | 0.014 |
| MEDP061 | N,N-Dimethylglycine | 1.208 | 3.189 | 0.401 | 0.006 |
| MEDP125 | Choline | 0.944 | 3.841 | 0.806 | 0.003 |
| MEDP405 | 3-Aminoisobutanoic Acid | 0.901 | 2.718 | 0.599 | 0.002 |
| MEDP853 | Choline chloride | 1.043 | 2.969 | 0.853 | 0 |
