# Supplementary figures and images for: Immune and physiological responses in Penaeus monodon to ammonia-N stress: a multi-omics approach
Source: Front Immunol. 2024 Dec 10;15:1510887. doi: 10.3389/fimmu.2024.1510887 (PMC11666502; doi:10.3389/fimmu.2024.1510887)

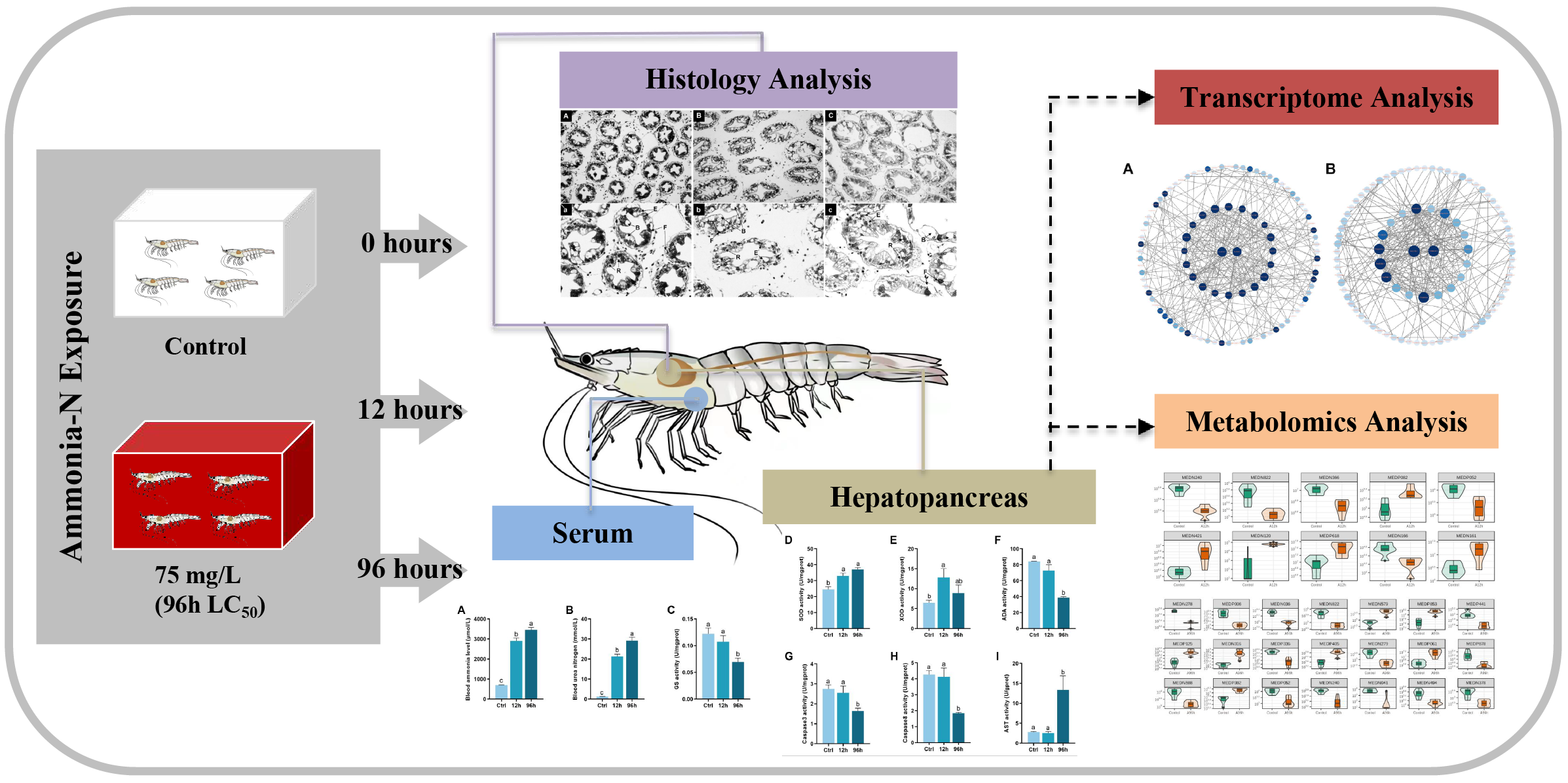

Supplement: Supplementary file 2 [file Image1.tif]
